# Development and biochemical characterization of freeze‐dried guava powder fortified with *Lactobacillus plantarum*

**DOI:** 10.1111/1750-3841.17537

**Published:** 2024-11-26

**Authors:** Ali Asad Yousaf, Hui Zeng, Kashif Sarfraz Abbasi, Teresa Bergholz, Muhammad Siddiq, Kirk Dolan

**Affiliations:** ^1^ Institute of Food & Nutritional Sciences PMAS‐Arid Agriculture University Rawalpindi Pakistan; ^2^ Department of Food Science & Human Nutrition Michigan State University East Lansing Michigan USA

**Keywords:** freeze drying, guava powder, *Lactobacillus plantarum* supplementation, physico‐chemical properties

## Abstract

Guava (*Psidium guajava* L.) is one of the most nutrient‐dense fruits, which is native to tropical and subtropical regions of the world. The processing of value‐added products from guava has not been carried out on a scale similar to some other fruits, which offers an opportunity to fully exploit the potential of this fruit, such as guava‐based nutraceutical food products. The objectives of the present study were to develop freeze‐dried guava powders (FDGPs) from two guava varieties (white and pink) and characterize their physico‐chemical and nutritional properties. FDGP was also incorporated with probiotic strains of *Lactobacillus plantarum*, to develop a healthy nutraceutical probiotic supplement. Functional groups assessed by Fourier transform infrared (FTIR) spectroscopy exhibited the existence of strong C–Br stretch, O–H stretch, and C = C stretch vibrations; however, scanning electron micrograms (SEMs) showed the flaky structure indicating the presence of starch, dietary fibers, and esterified groups of pectin. Significant mineral concentrations (mg/100 g) of potassi‐um (323–362), magnesium (26.2–28.8), zinc (0.43–0.51), and iron (0.52–0.63) were observed in FDGPs. The FDGP samples from both guava varieties had high levels of crude fiber (43.94–46.29%), vitamin C (2.27–2.49 mg/g), and phenolic compounds (57.50–61.86 mg GAE/g) as well as significant antioxidant properties. Fortification of FDGP with *L. plantarum* strains produced significant results in terms of probiotic viability that was nearly maintained at 10^8^ CFU/g up to 60 days in the final product. The viability of probiotics proved that FDGP is a good carrier of prebiotics and can be utilized as a potent probiotic supplement.

## 1 I‐NTRODUCTION

Guava (*Psidium guajava* L.: Myrtaceae) is a popular tropical fruit due to its distinct flavor, aroma, and high nutritional value. It is loaded with minerals, vitamins, antioxidants, and dietary fiber, making guava as an ideal fruit for a healthy diet. Guava fruit typically contains 77–84% moisture, 8–12% carbohydrates, 1.9–2.2% proteins, 2.8–5.5% fiber, 0.6–0.8% crude fat, and 0.5–0.7% ash contents (Kadam et al., 2012; Yousaf et al., 2020). Guava fruit also possesses significant amounts of nutraceutical substances such as flavonoids (81–154 mg quercetin equivalents [QE]/100 g dry weight [dw]) and phenolic compounds (94–190 mg GAE/100 g dw) (Flores et al., 2015; Yousaf et al., 2020). Moreover, it can offer four times more vitamin C as compared to oranges (Kumari et al., 2013). Different pharmacological investigations have shown guava's anti‐diarrheal (Zulfiana & Fatmawati, 2023), anti‐diabetic (Díaz‐de‐Cerio et al., 2017), antimicrobial (Sharma et al., 2017), hepatoprotective (Zhu et al., 2020), anti‐allergic (Pathak, 2020), anti‐spasmodic (Bouchoukh et al., 2019), anti‐inflammatory (Gollaz‐Machuca et al., 2021), properties besides being found to be equally helpful in managing cardiovascular disorders (Singh et al., 2022). Due to these characteristics, nutritionists categorize guava fruit as a “super fruit” and frequently call it the “poor man's apple of the tropics” (Kaur, 2020). India, Pakistan, China, Brazil, Mexico, Indonesia, Malaysia, Thailand, and Vietnam are the topmost producers (Food and Agricultural Organization of the United Nations [FAO], 2022). More than 400 guava varieties exist around the globe, but only a few of them are commercially grown (Mathiazhagan et al., 2023).

In spite of the fact that guava can be processed into a number of products, including jams, jellies, marmalades, nectars, and juices, yet it is often consumed as fresh fruit. However, the quality of fresh fruit deteriorates quickly because guava falls under climacteric fruits, having high respiration rate and carrying active metabolism that limits its storage stability up to 2–3 days at ambient temperature (Nair et al., 2018). Furthermore, it is estimated that approximately 30–50% of guava fruit is annually wasted due to lack of proper postharvest management practices (Sultana et al., 2021). Value addition in the form of a nutraceutical food product would be a preferable choice to decrease postharvest losses and improve storage stability of this nutrient‐dense fruit. In recent times, there has been a notable surge in the global market for functional foods as well as the consumer desire to lead a healthy lifestyle. With 6.6% compound annual growth rate, the functional food ingredients market is anticipated to increase to $94.21 billion by the end of 2023 (Dable‐Tupas et al., 2020). Aside from the availability of conventional food products, the guava fruit offers enormous potential for the development of nutraceutical/functional foods with enhanced health benefits. Nowadays, the food industry is focusing toward the production of fruit‐based dehydrated food items with the aim to maximize nutrients’ preservation with proven health benefits. In this regard, the production of fruit powders is potentially an ideal technique not only to retain nutritional and sensory quality but also to increase the consumption of the fruits on regular basis (Nunes et al., 2016).

Traditional drying methods, like air‐drying or sun‐drying, can sometimes result in shriveled or toughened fruit with altered flavors. However, freeze drying has been shown to be an efficient technique to produce fruit powders with optimal nutritional quality (Harguindeguy & Fissore, 2020). Freeze‐dried fruit powders have gained substantial interest in the scientific community due to their potential applications in the food industry, nutraceuticals, and functional foods. Researchers have explored the potential of using freeze‐dried fruit powders as a source of bioactive compounds for developing nutraceutical products. These products are designed to have health benefits beyond their basic nutritional value. For example, probiotics, like *Lactobacillus plantarum*, can be added to freeze‐dried guava powder (FDGP) to enhance its health benefits such as prevention and treatment of gastrointestinal disorders (del Campo et al., 2014), reducing inflammation (Lee et al., 2014), boosting the immune system (Kumar et al., 2017), and managing hyperlipidemia (Miremadi et al., 2016; Teng et al., 2020). Therefore, to grasp aforesaid benefits, development of a fruit‐based food product with enhanced nutraceutical attributes and a longer shelf life would be essential. FDGP supplemented with *L. plantarum* would thereby offer consumers with a handy and healthful nutraceutical product. The main objectives of present study were to develop FDGP from two guava varieties (white and pink flesh) and evaluate its physico‐chemical and nutritional properties. Additionally, the survivability of probiotic strain *L. plantarum* incorporated into the FDGP and during subsequent storage was also assessed.

## 2 MATERIALS AND METHODS

### 2.1 Preparation of fruit samples

The fresh guava fruits, white (cv. Gola) and pink (cv. Semenyih), were purchased from Tropical Fruit Box^®^. The fruits were washed/sanitized with sodium hypochlorite solution (1% w/v) and cut into 1 × 1 × 1 cm cubes and deseeded. Thus, prepared guava cubes were sealed in high density polyethylene (HDPE) and stored at −18°C until further processing. All analytical grade chemicals and reagents were procured from Thermo Fisher Scientific.

### 2.2 Freeze drying

FDGPs were obtained by following the method of Verma et al. (2015) with some modifications. The fruit cubes were placed in a lab‐scale freeze dryer (Harvest Right) at −55°C with 550 mTorr vacuum pressure for 18–22 h. After complete drying, the fruit samples were grounded and filtered through US 20 mesh size (0.841 mm) sieve to obtain uniform particle size powder. The moisture content of FDGP was in the range of 4.2–4.5%. The FDGP was then sealed packed in HDPE bags and stored in refrigerator (5–7°C) for further analysis.

### 2.3 Physical analysis

#### 2.3.1 Moisture contents and water activity

Moisture contents of FDGP were determined through an electronic moisture balance (Sartorius MA30) at 105°C (Aragüez‐Fortes et al., 2019). Water activity (a_w_) of FDGP was measured by using a bench top water activity meter (AquaLab TDL 2, Meter Group Inc.). The FDGP samples were put in the clear sample cups that were inserted into the sample port at 25°C (Lee et al., 2022) and recorded the *a*_w_ reading.

#### 2.3.2 Bulk density

The bulk density of the FDGP samples was determined according to the method of Verma et al. (2015). For the purpose, 2.5 g of FDGP was poured into a 10 mL graduated measuring glass cylinder. The ratio of the powder's mass to its volume of occupied space was used to compute the bulk density, as g/cm^3^.

#### 2.3.3 Color measurements

The color of FDGP was measured using a Hunter Lab colorimeter, model Color flex EZ (HunterLab), and the results were reported as L* (lightness), a* (redness), and b* (yellowness) coordinates.

#### 2.3.4 SEM morphology of FDGP

The morphology of the FDGP samples was analyzed by scanning electron microscopy technique (SEM) according to the method of Domin et al. (2020). The powdered samples were first mounted on aluminum stubs using a double‐sided adhesive tape followed by gold coating and then examined under scanning electron microscope (SEM‐Tescan MIRA 2, CZ) operating at 5 kV accelerating voltage and 80 torr vacuum.

#### 2.3.5 Functional group analysis

Fourier transformed infrared spectroscopy (FTIR) was used to determine the major functional groups present in the FDGP samples. The powder samples were first gently homogenized with potassium bromide (KBr) powder and then compressed into pellet form by using a tablet presser. The samples were run under Bruker Alpha Model FTIR with wavelength range of 4000–400 cm^−1^ (Athmaselvi et al., 2014).

### 2.4 Nutraceutical analysis

Nutraceutical compound estimation was carried out according to the procedures followed by Yousaf et al. (2020). The FDGP extracts were obtained by taking 20 g of FDGP samples (separately) in 500 mL conical flask, and then 200 mL of 80% methanol (80:20 methanol–water v/v) was mixed. The mixing was further carried out in an orbital shaker overnight at 25°C. The resultant homogenous mixture was separated from residues by using Whatman No. 1 filter paper. Then the filtrate was further concentrated through rotary evaporator under reduced pressure at temperature of 45°C. The concentrated extracts were weighed and refrigerated until used for further analysis.

#### 2.4.1 Total phenolic content

Total phenolic compounds were determined by using Folin–Ciocàlteu (FC) reagent according to the method of Matić et al. (2017) with slight modifications. The extract (0.5 mL) was transferred into a 25 mL volumetric flask and mixed with 5 mL of 2 N FC reagent. Then 4 mL of 7.5% sodium carbonate solution was added to the previous mixture. After that, 80% methanol was added to fill the total volume of the flask. The mixture was then wrapped in aluminum foil for 1 h (darkness). Meanwhile, standard solutions of gallic acid of different concentrations (50–450 ppm) were prepared to quantify the total phenolic contents. The absorbance of each sample was recorded at 765 nm using spectrophotometer, and a standard curve was drawn in MS Excel. Similarly, after 1 h the absorbance of sample solution was also taken. Quantification was carried out using regression equation obtained from standard curve. The TPC are reported as mg GAE/g, on dw basis.

#### 2.4.2 Total flavonoid contents

Total flavonoid contents (TFC) were measured by a spectrophotometric method as described by Matić et al. (2017). In a 10 mL volumetric flask, 1.0 mL of aqueous extract was poured through pipette. Then 5 mL of double distilled water was added in the same flask. After the addition of distilled water, 0.3 mL of 5% sodium nitrite (NaNO_2_) solution was added. After 5 min, the previous mixture was supplemented with 0.6 mL of 10% aluminum chloride (AlCl_3_) solution. Again, after 5 min, 1.0 M sodium hydroxide (NaOH) solution (2 mL) was added. Lastly, the volume of flask was made to 10 mL with distillated water. Standard solutions of varying levels (50–450 ppm) was made by using quercetin as standard compound and absorbance were noted at 510 nm on UV–visible spectrophotometer. From obtained readings, a standard curve was drawn in MS‐Excel and TFC were reported as mg of QE, as mg QE/g on dw basis.

#### 2.4.3 Radical scavenging assay

For the estimation of radical scavenging activity (RSA), DPPH (diphenyl picrylhydrazyl) was used according to the method of Verma et al. (2018). A 0.1 mM solution of DPPH was prepared using 80% methanol. From that solution, 3.9 mL was taken in cuvette and with the help of micro‐pipette, 0.1 mL of fruit extract was added, and absorbance was documented at 517 nm using UV–visible spectrophotometer. After that, the cuvette was placed in dark for 30 min, and the absorbance was noted again. RSA was determined by using the below cited equation and expressed as the difference in absorption of the testing sample responsive to the control sample (0.1 mM DPPH solution without the extract):

$$\mathrm{DPPH}\;\mathrm{RSA}\left(\%\right)=\left(A_{0}-A_{1}\right)/A_{0}\times 100$$

where *A*_0_ and *A*_1_ were absorbances of the control and the treatment sample, respectively.

#### 2.4.4 Ascorbic acid (as vitamin C)

Ascorbic acid, as mg/g of vitamin C, was determined by using Tillman's reagent (2,6‐dichlorophenolindophenol sodium salt solution) according to Association of Official Analytical Chemists (AOAC, 2016) method No. 967.21. A 10 g of FDGP was taken in a beaker (500 mL) whose volume was brought up to 100 mL with 3% metaphosphoric acid. Then 150 mL of distilled water was added and waited for 30 min. After that, the solution was filtered, and 10 mL of filtrate was titrated with the Tillman's reagent (dye) until distinct rose‐pink color persist for more than 5 s (end point). The ascorbic acid contents were quantified by the standard equation described in AOAC (2016), and reported as mg/g on dw basis.

#### 2.4.5 Crude fiber

Crude fiber was determined according to AOAC (2016) method No. 978.10. Fruit sample of 2 g was first dried and defatted, then poured into beaker followed by digestion under heat with 1.25% sulfuric acid solution for 30 min. After heating, mixture was filtered and washed thoroughly with water to make it free of acid. This was followed by heating with 1.25% sodium hydroxide solutions again for 30 min. The filtrate was washed with distilled water, and residues were transferred to the pre‐weighed crucibles and ignited at 550°C in a muffle‐furnace for 2–3 h until white ash formation. The crude fiber was calculated according to the following equation:

$$\begin{array}{ccc} & & \mathrm{Crude}\;\mathrm{fiber}\left(\%\right)=\\ & & \frac{\mathrm{weight}\;\mathrm{of}\;\mathrm{dried}\;\mathrm{fiber}-\mathrm{weight}\;\mathrm{of}\;\mathrm{residual}\;\mathrm{ash}}{\mathrm{weight}\mathrm{of}\mathrm{sample}}\times 100 \end{array}$$

#### 2.4.6 Mineral analysis through ICP‐MS

Mineral contents were analyzed using inductively coupled plasma mass spectrometry (ICP‐MS). For ICP‐MS analysis, the acid digestion of FDGP samples was carried out by following the method of Uddin et al. (2016). To 0.5 g dried sample, 5 mL of 65% HNO_3_ was added and heated to 90°C in water bath for approximately 2 h. After that, 2.5 mL of HNO_3_ of the same concentration was added followed by further boiling (∼45 min) until a clear solution was obtained. The samples were then diluted 1000 times with HPLC grade ultra‐pure water. The quadrupole inductively coupled plasma mass spectrometer (Thermo Fisher Scientific Inc.) was used for mineral estimations according to the procedures employed by Khan et al. (2014). The ICP‐MS was combined with a Photon Machines Analyte G2 193 nm excimer laser ablation system equipped with a 15 × 15 cm^2^ HelEx sample cell. The operating conditions were depicted in Table 1.

**TABLE 1:** Operating conditions for inductively coupled plasma mass spectrometry (ICP‐MS).

Operating conditions	Details
*Plasma conditions*:	
RF generator	Frequency = 10 MHz, power output = 1.3 kW
Acquisition mode	Spectrum
Integration time	0.3/S
Spray chamber temperature	2.0°C
Sample uptake rate	0.1 mL/min
Carrier Ar flow rate	1.0 L/min
Oxide	(CeO/Ce) < 1.0%
Double charge	(Ce^++^/Ce) < 3.0%
Number of readings per replicate	3
*Interface*:	
Sampling depth	6.5 mm
Vacuum	Interface:4 torr, quadrupole: 2 × 10^−5^ torr
Sampler cone	Nickel, 1.0 mm orifice diameter
*Analytical masses*:	^23^Na, ^24^Mg, ^27^Al, ^39^K, ^44^Ca, ^48^Ti, ^51^V, ^55^Mn, ^53^Cr, ^57^Fe, ^59^Co, ^60^Ni, ^63^Cu, ^66^Zn, ^111^Cd, ^118^Sn, ^133^Cs, ^137^Ba, ^208^Pb

### 2.5 Supplementation of FDGP with *Lactobacillus plantarum*

Supplementation of probiotic strain into FDGP was carried out using the procedure of Borges et al. (2016) with some modifications. For this purpose, the probiotic strain of *L. plantarum* (*Lactiplantibacillus plantarum* ATCC 14917) was obtained from ATCC (The Global Bioresource Center) and stored at −80°C in MRS broth (Dot Scientific Inc.) supplemented with 15% (w/v) glycerol. *L. plantarum* was inoculated into 5 mL of sterile MRS broth from freezer stock and incubated at 37°C for 24 h in an anaerobic jar with AnaeroPack (Mitsubishi Gas Chemical America Inc.) to achieve an anaerobic environment. The culture was then inoculated at the level of 2% (v/v) in a second MRS broth for another 24 h at 37°C anaerobically. To obtain concentrated culture pellets, the liquid culture was centrifuged at 5184 RCF for 7 min at 4°C. After centrifuging, for each bottle of 250 mL culture liquid, MRS broth was removed by decanting supernatant and suspending pellets in 50 mL of 0.85% (w/v) NaCl solution, and vortexing the NaCl solution with culture pellets for 2 min to homogenize. The culture liquid was centrifuged at 5184 RCF for 7 min at 4°C. The supernatant was decanted, and for each 250 mL of supernatant, 8 mL of cryoprotectant solution composed of 5% (w/v) sucrose and 5% (w/v) trehalose was added. *L. plantarum* was freeze dried with the cryoprotectant mix at −80°C for 18–24 h using Harvest Right Scientific Model Freeze Dryer.

The freeze‐dried *L. plantarum* powder was homogenized with FDGP at 10% (w/w). A non‐inoculated guava powder sample (3.0 g) was used for plating onto plate count agar (Dot Scientific Inc.) to quantify the natural microbes present in the FDGP. The mixed dry powder samples were stored anaerobically in sealed plastic bags at room temperature for bacterial enumeration test on the day 0, 6, 12, 18, 24, 30, and 60. For that purpose, 0.5 g supplemented FDGP was added to 50 mL sterile water, vortexed, and held at room temperature for 1 h. Then the mixture was again vortexed and serial dilutions were made with phosphate‐buffered saline, and plating was done using MRS agar plates. The MRS agar plates were incubated in an anaerobic jar for 24–48 h at 37°C to facilitate bacterial growth. The colonies on plates were counted and recorded by Interscience Scan 500 Colony Counter. For each time point, three replicates of guava powder were sampled.

### 2.6 Statistical analysis

Data were presented in triplicate with mean and standard deviation. Significant differences (*p* < 0.05) within means were analyzed by analysis of variance and Tukey's test by using Minitab statistical software, version 18.

## 3 RESULTS AND DISCUSSION

### 3.1 Physical properties

FDGPs from white and pink varieties were characterized according to their physical attributes (moisture, water activity, bulk density, color measurements), as presented in Table 2., Results showed that both the FDGP had low moisture contents (4.12–4.52%) as well as low water activity (0.29–0.35). The physical state and rate of reconstitution of powdered foods are significantly influenced by the moisture content (Saifullah et al., 2016). Moreover, this was a clear indication that the freeze drying considerably reduced the water contents of fresh guava fruits, thus making FDGP less prone to microbial proliferation. Typically, at water activity of <0.6, a food product becomes microbiologically stable and shows no growth of spoiling organisms or pathogens (Rahman & Labuza, 2007).

**TABLE 2:** Physical analysis of freeze‐dried guava powders (FDGPs) from white and pink varieties.

Parameters	FDGP (white)	FDGP (pink)
Moisture (%)	4.12^b^ ± 0.23	4.52^a^ ± 0.31
Water activity (a_w_)	0.29^b^ ± 0.01	0.35^a^ ± 0.04
Bulk density (g/cm^3^)	0.36^a^ ± 0.12	0.39^a^ ± 0.08
Color measurements:		
L*	84.91^a^ ± 1.05	75.12^b^ ± 1.02
a*	−0.61^b^ ± 0.02	13.05^a^ ± 0.02
b*	21.38^b^ ± 0.19	37.54^a^ ± 0.21
Chroma	21.39^b^ ± 0.11	39.74^a^ ± 0.13
Hue angle	92.79^a^ ± 2.53	69.91^b^ ± 2.77

*Note*: Values are given as mean ± SD. Means sharing different letters in the same row are significantly different (Tukey's HSD test, *p* ≤ 0.05).

The bulk density of FDGP was found to be in the range of 0.36 to 0.39 g/cm for FDGP from white and pink flesh guava, respectively. Koc et al. (2008) demonstrated that the drying process in freeze drying was the result of sublimation, leaving cavities inside the solid particles without much shrinking and thus producing better quality dried fruit solids. Similarly, Osorio et al. (2011) illustrated that much lighter fruit powder could be prepared through freeze drying as compared to other traditional drying techniques.

The instrumental (tristimulus) color measurements of dried food products are vital quality indicators in terms of a product's sensory appeal and overall consumer acceptance (Quek et al., 2007). Table 2 presents color values for white and pink FDGPs which revealed that both the powders were brighter in color, as high luminosity (L*) values of 84.91 and 75.12, respectively. However, white flesh FDGP was more luminous than that of pink flesh FDGP. However, taking into account a* and b* color coordinates (redness and yellowness), the pink FDGP showed positive a* and b* values (+13.05 and +37.54, respectively), suggesting the location of these color parameters in the first quadrant and being associated with characteristic pink coloration of the FDGP. Nevertheless, white flesh FDGP that depicted negative a* value (−0.61) and positive b* values (+21.38), showing a creamy white color with greenish tint. Furthermore, chroma values demonstrated the third dimension of color quadrant that shows intensity or purity of color. It was observed that pink FDGP had more intense in color than that from white guava. Hue angle is another color property that distinguishes red, yellow, green, and blue colors, and it is, therefore, dependent upon a* and b* color coordinates. In the present study, the white flesh FDGP had higher hue angle values than the pink one due to the deviations among the other color coordinates.

The variations in the values of a* and b* coordinates have been reported to be associated with phenolic content of the plant‐based powders. While studying sweet potato powder, Ahmed et al. (2010) demonstrated that the redness and yellowness were correlated with the total phenol level of spray dried sweet potato powder. Furthermore, Shishir et al. (2017) reported similar results that luminosity, redness, and hue angle are likely associated to lycopene contents of pink guava powder. The results of our investigation were similar to those reported by Costa et al. (2009), Caliskan et al. (2015), Verma et al. (2015), Saifullah et al. (2016), and Frabetti et al. (2021). These findings served as a crucial springboard for characterizing guava fruit powders and consequently producing value‐added nutraceutical food products from them.

#### 3.1.1 Morphology of FDGP using SEM

**FIGURE 1:**
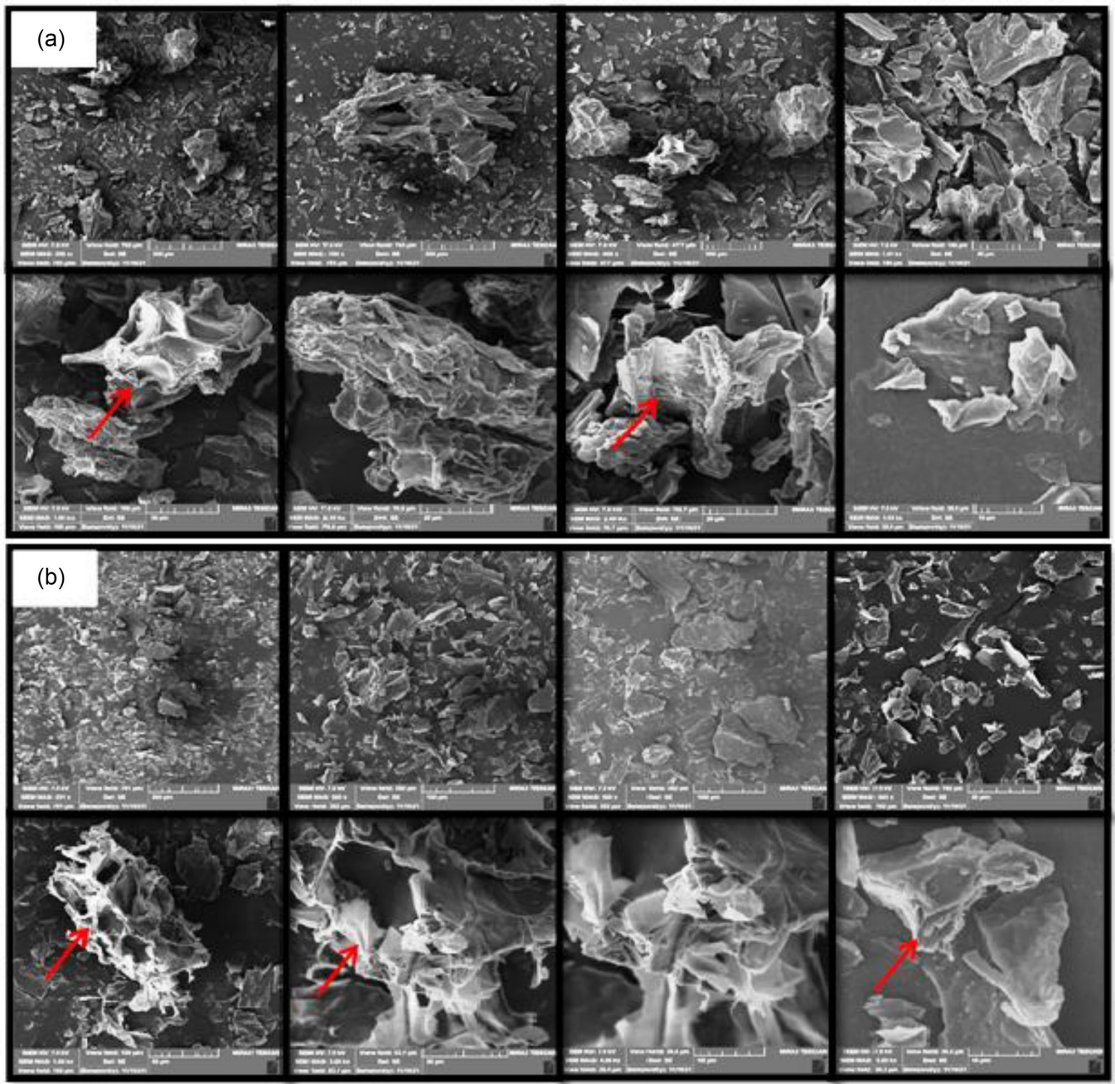
Scanning electron microscopy of (a) white and (b) pink freeze‐dried guava powders (FDGPs) at 250×–5000× magnification. Arrows indicate pectin layers.

Scanning electron micrographs (SEM) of white and pink FDGPs are shown in Figure 1a,b, respectively, which exhibited flake‐like structures, with irregular size and shape. Both white and pink FDGP appeared to be identical with respect to size distribution and particle morphology.

Because the powders were made by freeze drying the whole fruit, therefore, it resulted in somewhat irregular shapes of powder particles exhibiting fibrous and porous characteristics. As reported by Zea et al. (2013), whole fruit powders are usually contained with a variety of hydrophilic fibrous substances, including fiber, sugar, protein, and fat, due to which complex and agglomerated particles formed. In morphological terms, two phases were observed in both guava solids: a lamellar surface structure (with flake form) and a spherical arrangement. These two morphologies suggested that the dehydration processes may have caused the solids to separate into different phases (Athmaselvi et al., 2014). Furthermore, the SEM of FDGPs also depicted pectin layers as the structural component on the surface of freeze‐dried powder that was also observed by Osorio et al. (2011) while studying the morphology of guava powders. It is noteworthy that Marques et al. (2007) suggested that the solid state of water protects the fundamental structure and limits changes in the shape of the finished product with less shrinkage during lyophilization. Similar elucidations were also given by Durigon et al. (2016) for tomato powders, as well as by Caparino et al. (2012) and Zotarelli et al. (2017) for mango powders. Our findings are also similar to those reported by Osorio et al. (2011), Conceição et al. (2016), and Frabetti et al. (2021).

#### 3.1.2 Functional group analysis

**FIGURE 2:**
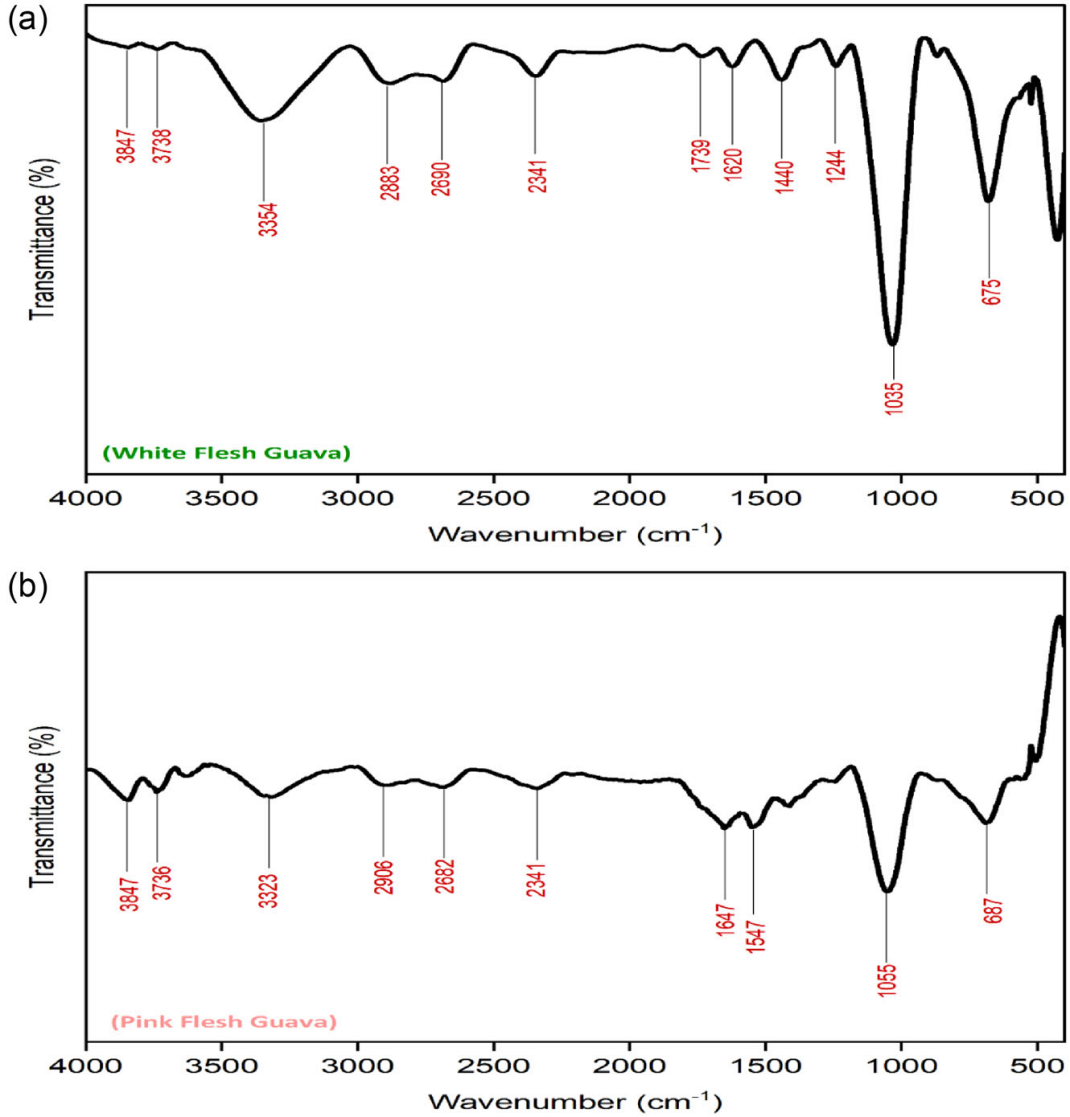
Fourier transformed infrared (FTIR) spectrum for freeze‐dried guava powder (FDGP) from (a) white and (b) pink guava varieties.

Functional group analysis in terms of FTIR spectroscopy of FDGP samples is depicted in Figure 2a,b. As shown in the FTIR graphs, both white and pink FDGPs were found to be identical; however, sharp peaks could be observed in white FDGP (Figure 2a). Peaks in the range of 700–500 cm^−1^ are assigned to C–Br stretch representing strong halo compounds (alkyl halides). Peaks at 1035 and 1055 cm^−1^ signified strong C–O and C–F bonding (the presence of esters and phenolic compounds) in the finger print region (Arahman et al., 2019). Anhydrides, carboxylic acids, esters, and alcohols also absorb in that region (i.e., 1440–1000 cm^−1^). The band ranges from 1200 to 950 cm^−1^ exhibited strong absorption by carbohydrates (Manrique & Lajolo, 2002). Peak at 1244 cm^−1^ illustrated medium C–N stretching (amines) as shown in Figure 2a for white FGDP. On the other hand, FTIR spectrum for pink FDGP exhibited peaks at 1547 and 1647 cm^−1^ indicating strong N–O stretching (nitro compounds) and strong C = C stretching (alkene group), respectively. The peaks at 2341 cm^−1^ were identical for both white and pink FDGPs (Figure 2a,b) showing strong O = C = O stretching (Xia & Shi, 2016). Peaks around 2600–3000 cm^−1^ for FDGP demonstrate medium C–H stretching vibrations of methylene. The region from 3700 to 3000 cm^−1^ relates to water and OH absorption frequencies (Zhang et al., 2001); this peak was observed in both white and pink FDGP. As discussed in SEM analysis, FTIR spectra (1200–950 cm^−1^) also confirmed the typical signals of polygalacturonic acid (pectic acid) and related pectic compounds. The observations noticed in the present study were in close agreement to the findings of Osorio et al. (2011) and Athmaselvi et al. (2014).

### 3.2 Nutraceutical evaluation of FDGPs

Nutraceutical potential of FDGP was evaluated in terms of fiber contents, vitamin C, total phenolic contents (TPC), total flavonoids content (TFC), and DPPH antioxidant activity. The presence of diversified bioactive substances in fruits is widely recognized as a fruit quality determining profile based on their intended consumption (Ali et al., 2011). The nutraceutical potential of guava fruits was exploited in a previous study by Yousaf et al. (2020) that provided the impetus for the present investigation. Table 3 summarizes the nutraceutical evaluation of FDGP that showed that dietary fiber was present in high concentration in guava powders with 43.94% and 46.29% concentration in white and pink FDGP, respectively.

**TABLE 3:** Nutraceutical properties of freeze‐dried guava powders (FDGPs) from white and pink varieties.

Parameters	FDGP (white)	FDGP (pink)
Crude fiber (%)	43.94^b^ ± 0.44	46.29^a^ ± 0.52
Total phenolic contents (mg GAE/g)	57.50^b^ ± 0.19	61.86^a^ ± 0.28
Total flavonoids (mg QE/g)	36.20^b^ ± 0.92	41.11^a^ ± 0.61
Antioxidant activity (%)	85.70^b^ ± 3.25	88.27^a^ ± 4.17
Ascorbic acid (mg/g)	2.27^b^ ± 0.17	2.49^a^ ± 0.14

*Note*: Values are given as mean ± SD. Means sharing different letters in the same row are significantly different (Tukey's HSD test, *p* ≤ 0.05).

The variation in fiber contents was most likely due to the varietal differences and growing conditions as well (Yusof, 2003). It is believed that the fruit‐based dietary fibers often possess superior nutritional quality than those of cereals owing to the presence of associated bioactive substances, lower value of metabolic energy as well as low phytic acid (an anti‐nutrient) contents (Vergara‐Valencia et al., 2007). These dietary fibers hold substantial contribution toward the prevention of chronic and degenerative diseases (Slavin, 2013). Because dietary fibers also serve as ideal prebiotics (Holscher, 2017), and the current analysis confirmed that FDGPs possessed significant concentration of dietary fiber; therefore, there are compelling reasons to market FDGP as a potent value‐added nutraceutical food product. The findings of our investigations are also in line with those reported by Costa et al. (2009), Osorio et al. (2011), Verma et al. (2013), and Nora et al. (2014).

Phenolics are one of the main classes of bioactive substances that act as principal antioxidants or free radical scavengers and have disease‐preventing and health‐promoting effects on human body (Han et al., 2018). The FDGPs from both white and pink varieties were found to be rich in phenolic compounds (Table 3). Significantly higher TPC and TFC were observed in pink FDGP (61.86 mg GAE/g, 41.11 mg QE/g, respectively), whereas 57.50 mg GAE/g (TPC) and 36.20 mg QE/g (TFC) were found in white FDGP. The carotenoids (β‐carotene, β‐cryptoxanthin) and anthocyanin (cyanidin chloride, malvidin 3‐glucoside, cyanidin 3‐glycoside) are typically responsible for higher values of phenolics (TPC, TFC) and typical reddish pink coloration of pink FDGP (Da Silva et al., 2014; Nora et al., 2014). Fruits dehydrated by the freeze drying may potentially facilitate the subsequent, higher extraction of phenolic components (Verma et al., 2015), mainly due to the fact that the phenolic compounds are localized in vacuoles at cellular level forming a defined structure. This structure disintegrates during the drying or dehydration process, releasing more phenolic compounds as well as oxidative and hydrolytic enzymes that may also degrade the phenolic compounds (Toor & Savage, 2006). Similarly, Samoticha et al. (2016) illustrated that freeze drying retained more polyphenolic substances than other drying methods.

Table 3 presents the ascorbic acid values for FDGP that ranged from 2.27 to 2.49 mg/g of white and pink fleshed FDGP, respectively. Similar results were also reported by Verma et al. (2015) who estimated ascorbic acid contents of guava fruit powder obtained from various dehydration methods. It was observed that freeze drying was the best method to obtain guava fruit powder, as minimum loss of vitamin C was observed during freeze drying of guava fruit followed by vacuum drying. This may be due to the fact that the rate of oxidation was slower in freeze drying as compared to vacuum, spray, and sun drying (Mishra et al., 2009). It is important to note that the guava fruit may contain 3–4 times as much vitamin C as an average sized orange fruit (Uddin et al., 2002).

In addition to significant concentration of vitamin C, our results also showed higher levels of radical scavenging (antioxidant activity) by both the FDGPs. The FDGP from pink variety showed 88% RSA, whereas white FDGP exhibited 85% RSA. There is always a strong positive correlation between phenolic compounds and % RSA; thus, higher the phenolics, the higher the probability of free radical scavenging ability (Abbasi et al., 2019; Yousaf et al., 2020). The findings of current investigation regarding antioxidant properties and bioactive compounds of guava powders are also comparable with those reported by Nora et al. (2014), Shishir et al. (2018), Tan et al. (2020), and Frabetti et al. (2021).

#### 3.2.1 Mineral content of FDGP

ICP‐MS has been successfully applied for the analysis of trace elements in a variety of food samples due to its well‐known benefits of sensitivity, specificity, and multi‐element detection (Ali et al., 2021; Nardi et al., 2009; Toncheva et al., 2016). For the purpose, the quantitative analysis of 20 mineral elements (Al, Na, Mg, K, Ca, Ti, V, Cr, Mn, Fe, Co, Ni, Cu, Zn, Cd, Sn, Sb, Cs, Ba, and Pb) was carried out after acid digestion of the FDGP samples. Table 4 presents the results pertaining to mineral quantification through ICP‐MS from FDGP. The data exhibited that the pink FDGP proved to be more mineral enriched than that of the white FDGP. However, FDGPs from both the white and pink varieties had significant quantities (mg/100 g) of essential minerals (Na: 23.73–26.79, Mg: 26.22–28.86, K: 322.98–361.62, Ca: 21.65–22.91, Fe: 0.52–0.63, Cu: 0.009–0.010, and Zn: 0.425–0.512). On the other hand, it was observed that the amounts of toxic trace elements (Al, Cd, and Pb) in FDGP were extremely low, demonstrating that there is no health risk associated with its use. FAO and the World Health Organization (WHO) have devised critical limits for essential minerals. According to these values, the dietary reference intakes for Zn, Cu, Fe, and Cr are 8–11, 0.7–0.8, 8–18, and 0.025–0.035 mg/day, respectively (WHO, 2004). Therefore, FDGP would be an excellent contributor to the consumers’ daily intake of vital minerals. The findings on the mineral composition of FDGP were also in strong accord with those reported by Verma et al. (2015). Similar results were also produced by Pereira et al. (2014) and Chiveu et al. (2019), who discovered guava fruit to be a substantial source of beneficial micronutrients.

**TABLE 4:** Mineral composition of freeze‐dried guava powders (FDGPs) from white and pink varieties (mg/100 g).

Elements	FDGP (white)	FDGP (pink)
Al	0.218 ± 0.08	0.193 ± 0.08
Na	23.73 ± 0.66	26.79 ± 1.13
Mg	26.22 ± 0.54	28.86 ± 1.24
K	322.98 ± 4.12	361.62 ± 4.82
Ca	21.65 ± 0.46	22.91 ± 0.84
Ti	<0.1	<0.1
V	<0.1	<0.1
Cr	<0.1	<0.1
Mn	<0.1	<0.1
Fe	0.52 ± 0.07	0.63 ± 0.04
Co	<0.1	<0.1
Ni	<0.1	<0.1
Cu	<0.1	<0.1
Zn	0.43 ± 0.08	0.51 ± 0.08
Cd	ND	ND
Sn	<0.1	<0.1
Sb	ND	ND
Cs	<0.1	<0.
Ba	<	<0.1
Pb	<0.1	<0.1

*Note*: Values are given as mean ± SD. ND = Not detected

#### 3.2.2 Supplementation of *Lactobacillus plantarum* to FDGP

**FIGURE 3:**
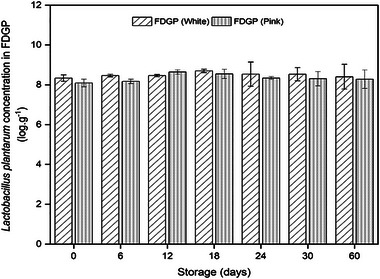
Probiotic enumeration/viability test of *Lactobacillus plantarum* in freeze‐dried guava powders (FDGPs) from white and pink varieties.

Figure 3 shows results regarding bacterial enumeration/viability of *L. plantarum* in FDGP. It was revealed that the viability of *L. plantarum* remained consistent in both white and pink FDGP samples, with no significant decline observed over a 60‐day storage period. On the initial day of inoculation, the white FDGP samples had a concentration of 8.33 ± 0.16 log CFU/g or log/g, and by day 60, it was 8.40 ± 0.62 log CFU/g. The pink FDGP sample started at 8.09 ± 0.19 log CFU/g and maintained a concentration of 8.27 ± 0.46 log CFU/g by day 60 of storage. This was in accordance with the recommended dosage for foods containing probiotic supplements, as estimated by Chaikham (2015) and Laličić‐Petronijević et al. (2015), who proposed that a minimum of 10^6^ CFU/g of active probiotics should be present in the food supplement prior to consumption. The current investigation also exhibited that freeze drying is as an effective method for preserving the viability of *L. plantarum* during extended storage at ambient temperatures. Although the survival of probiotics in a food matrix is greatly influenced by moisture content, due to severe temperature and humidity fluctuations, conventional fruit drying methods offer lower water contents than that of freeze drying, which may be the cause of higher survival rate of probiotics in freeze‐dried powders. Furthermore, the survival of probiotic culture in food matrix is primarily reliant on the inoculation technique used. In comparison, the freeze‐drying approach for supplementation of probiotic culture to fruit powder exposed better cell viability than incorporating in fruit juice at refrigeration temperatures. In our current investigation, the probiotic (*L. plantarum* ATCC 14917) and guava fruit were separately freeze‐dried before being mixed, which yielded higher cell viability during extended storage. This aligns with the findings of Borges et al. (2016), who recorded significantly reduced viability of *L. plantarum* during storage when fresh fruits (apple, banana, and strawberry) were immersed in *L. plantarum* culture prior to simultaneous drying. Aryaee et al. (2023) also recommended freeze‐drying method for the production of high‐quality fruit powders fortified with probiotics. Furthermore, the outcomes of our research are in agreement with prior studies by Borges et al. (2016) and do Espírito Santo et al. (2012), who emphasized the positive impact of fiber content on probiotic cell viability. In the comparative assessment, guava, with its higher fiber content relative to banana, apple, and strawberry, demonstrated superior *L. plantarum* survival rates.

## 4 CONCLUSION

Fruit powders are among the nutrien‐tdense foods that are becoming increasingly popular among consumers. The findings of our study have demonstrated that FDGPs, which were high in vitamin C, total phenolic compounds, dietary fiber, and key minerals, may be used as a component in the development of value‐added food product. The development of value‐added food products, such as FDGP supplemented with *L. plantarum*, seems to be an effective way to integrate the health benefits of probiotic cultures with fruits. Probiotic viability has shown that FDGP is an efficient prebiotic carrier and may be utilized as a potent probiotic supplement. The utilization of these fruit powders can have more economic and commercial potential as a reliable source of nutraceuticals with added health advantages. In future, further advances in micro‐encapsulation technology should allow for even higher survivability of probiotics in fruit powders that can be utilized as functional foods, personalized nutrition, and sustainable, clean‐label food products.

## AUTHOR CONTRIBUTIONS

**Ali Asad Yousaf**: Conceptualization; methodology; investigation; formal analysis; writing—original draft; writing—review and editing. **Hui Zeng**: Methodology; formal analysis; writing—review and editing; investigation. **Kashif Sarfraz Abbasi**: Conceptualization; supervision; writing—review and editing. **Teresa Bergholz**: Writing—review and editing; resources. **Muhammad Siddiq**: Writing—review and editing; supervision. **Kirk Dolan**: Supervision; resources; writing—review and editing.

## CONFLICT OF INTEREST STATEMENT

The authors declare that they have no known conflicts of interest or personal relationships that could have appeared to influence the work reported in this paper.

## Data Availability

The data that support the findings of this study are available on request from the corresponding author. The data are not publicly available due to privacy or ethical restrictions.
